# Ruxolitinib Controls Lymphoproliferation and Diabetes in a STAT3-GOF Patient

**DOI:** 10.1007/s10875-020-00864-w

**Published:** 2020-09-17

**Authors:** Oliver Wegehaupt, Tina Muckenhaupt, Matthew B Johnson, Karl Otfried Schwab, Carsten Speckmann

**Affiliations:** 1 Faculty of Medicine, Center for Pediatrics and Adolescent Medicine, Medical Center, University of Freiburg, Mathildenstr 1, 79106 Freiburg, Germany; 2 Faculty of Medicine, Center for Chronic Immunodeficiency (CCI), Medical Center - University of Freiburg, Institute for Immunodeficiency, University of Freiburg, Freiburg, Germany; 3 Diabetes Centre, Center for Pediatrics and Adolescent Medicine, Reutlingen, Germany; 4 Institute of Biomedical and Clinical Science, University of Exeter Medical School, Exeter, UK; 5 Faculty of Medicine, Center for Pediatrics and Adolescent Medicine, Division of Pediatric Diabetes, Medical Center, University of Freiburg, Freiburg, Germany

To the Editor

We report on a 2 9-/12-year-old male with STAT3-GOF mutation, whose exacerbation of neonatal-onset diabetes, lymphoproliferation, and autoinflammation was successfully treated with ruxolitinib.

The patient was born at term as the first child of non-consanguineous Caucasian parents and developed an insulin-dependent diabetes (IDDM) at the age of 3 months, initially presenting with polydipsia, fatigue, and vomiting. Laboratory findings showed pronounced ketoacidosis (pH 7.09; pCO2 28 mmHg, base excess − 25 mmol/l; ketone 4.9 mmol/l; blood glucose 32.3 mmol/l; HbA1c 5.6%; C-peptide 0.05 nmol/l; insulin 1.3 mU/l). Clinical investigation and initial abdominal ultrasound were unremarkable, i.e., without signs of lymphoproliferation. Ahead of diabetes therapy, insulin-autoantibodies (IAA; 20.6 U/ml) and glutamate-decarboxylase autoantibodies (anti-GAD; 1.1 kU/l) were positive. Genetic testing, initiated because of neonatal-onset IDDM, did not reveal any variants in *KCNJ11*, *ABCC8*, or *INS*. At initial discharge, the child was provided with sensor-assisted pump therapy and showed a moderate daily insulin demand of 0.5 IE/kg/day.

Between the age of 14 and 17 months, the patient developed additional autoimmune phenomena with progressive panniculitis at the anterior feet and accompanying lymphedema and lymphangitis (Fig. 1a). Additionally, marked inguinal lymphoproliferation was noted. Blood counts remained unremarkable. The child developed lipoatrophic skin lesions in areas where insulin was injected subcutaneously.

**Fig. 1:**
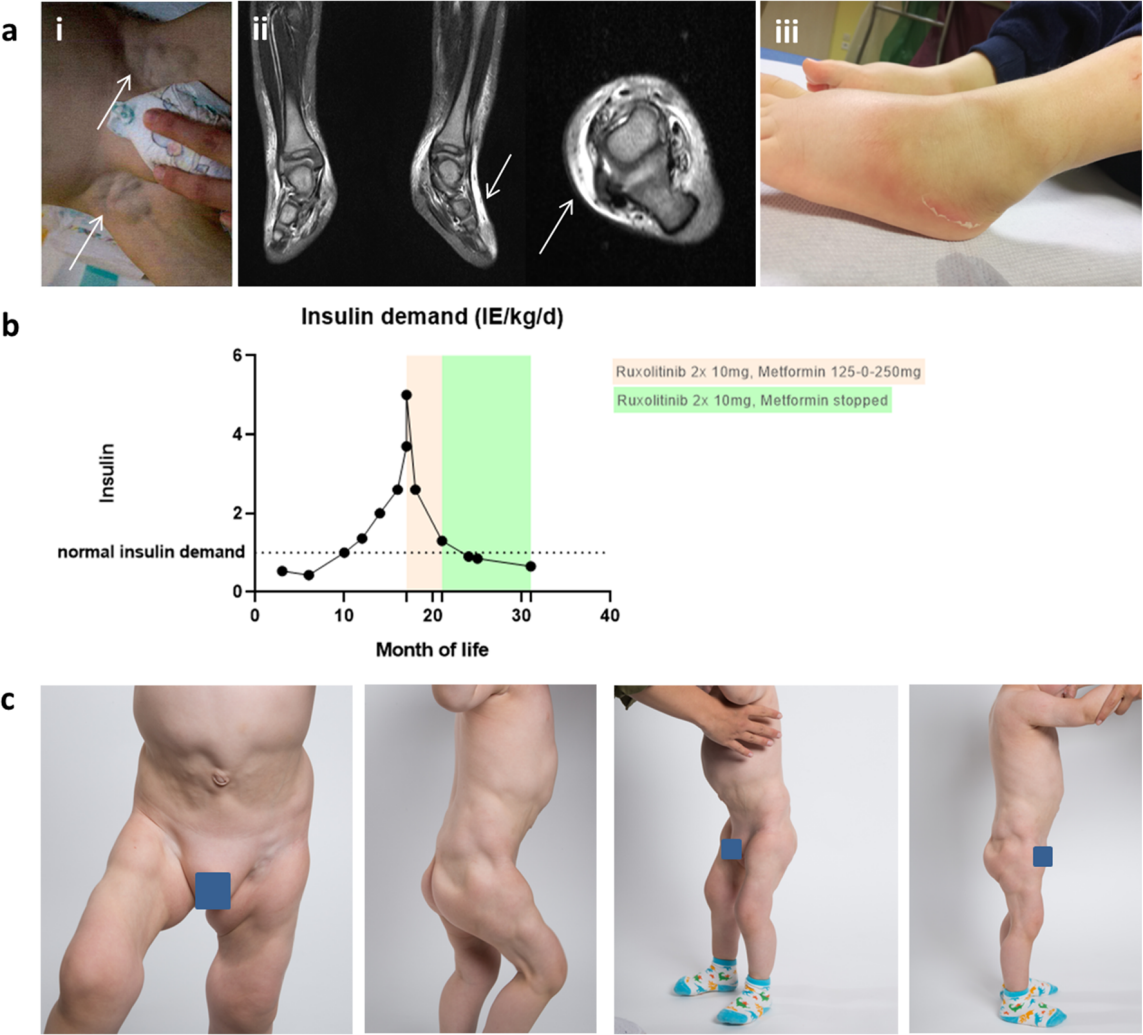
**a** Additional autoimmune-lymphoproliferative symptoms: (i) Pronounced lymphoproliferation observed at 17 months of life. (ii) Coronal and transversal T2-weighted magnetic resonance imaging showing autoimmune panniculitis as signal enhancement of subcutaneous fatty tissue at the ankles and the back of the feet. There is no significant involvement of muscles or tendons. (iii) Panniculitis at the back of the patient’s feet. **b** After diagnosis of neonatal-onset diabetes (fourth month of life), an increasing insulin demand up to a maximum of 6 IU/kg/day was observed. After the initiation of ruxolitinib and metformin therapy (from the 17th month of life, in red), the insulin demand dropped to normal levels of 0.6 IU/kg/day (normal range for children with type 1 diabetes in remission around 0.8–1.2 IU/kg/day). The low insulin demand remained stable under ruxolitinib monotherapy (from 21 months of age, in green). **c** Lipodystrophy down to the muscle fascia is pronounced in those areas where insulin has been administered previously. In places without prior application of insulin, a reconstruction of subcutaneous fatty tissue is evident under ruxolitinib therapy

At the age of 17 months, further genetic testing at the University of Exeter, which included 26 known genetic causes of neonatal diabetes (Twist BioScience custom targeted panel, Illumina NextSeq), identified a known pathogenic heterozygous gain-of-function (GOF) variant in *STAT3* (Exon 22; c.2144C > T, p.Pro715Leu). Parental testing confirmed the variant had arisen de novo, and no other rare variants were identified. The four previously reported patients with this variant presented with cytopenia (*n* = 4), signs of lymphoproliferation (*n* = 4), and also IDDM (*n* = 1) (supplementary Table 1).

Shortly after the genetic diagnosis, insulin demand rapidly increased to 5 IU/kg/day (normal range around 0.8–1.2 IU/kg/day) [1]. Even temporary use of continuous intravenous therapy did not improve insulin demand.

At arrival at our department, the patient was in a good overall condition, without acute or chronic infections, i.e., no signs of persistent viral infections. Prominent inguinal and abdominal lymphadenopathy was observed as well as marked general lipodystrophy. No splenomegaly was detected. Laboratory findings showed elevated IAA (> 22,000 nU/ml). Anti-tyrosine-phosphatase autoantibodies (IA2; 63 mU/ml) and anti-GAD (< 0.7 U/ml) were normal. Additional screening workup included negative results for thyreoperoxidase (TPO), thyroglobulin, adrenal cortex, zinc transporter 8, transglutaminase, and gliadin autoantibodies as well as for ANA and ANCA. Signs of inflammation (IgG 1159 mg/dl; soluble IL-2R 2876 U/mL) were observed. Even though insulin therapy was switched from insulin analogs to human insulin in order to avoid rise in anti-insulin autoantibodies, the insulin demand further increased to a maximum of 6 IU/kg/day. Metformin (250–0–125 mg) was added to decrease insulin-demand but was ineffective.

Based on the reported positive effects of ruxolitinib on lymphoproliferative and inflammatory manifestations in STAT3-GOF patients [2], we hypothesized that control of inflammation by this nonselective JAK1/2 inhibitor may also bear a potential to improve diabetes control in our patient. Ruxolitinib was started as an off-label therapy at an initial dosage of 15 mg/m^2^/day and increased to 40 mg/m^2^/day within 2 weeks. Indeed, we observed an immediate and significant drop in insulin demand. Metformin was discontinued after 4 months, and insulin demand remains to be very low (0.6 IU/kg/day) on ruxolitinib monotherapy for more than 1 year now (Fig. 1b). Lasting diabetes control was further documented by a stabilized HbA1c of 6.8%. IAA antibodies continuously decreased to 100 U/ml 8 months and 87.6 U/ml 15 months after start of JAK1/2 inhibition. The C-peptide remained low at 0.05 nmol/l at all times. Regulatory T cells (determined as CD25highFOXP3+ of CD4+ T cells) were at the lower limit for age (2.6%, normal range 2–9.6%), yet with a reduction of very CD25high CD4+ cells, possibly pointing towards an impaired differentiation of these cells. This observation was unaffected by ruxolitinib treatment (data not shown). Treg function was not assessed.

Follow-up appointments also showed maintained resolution of panniculitis and lymph node swelling. The lipodystrophy/atrophy of the subcutaneous fatty tissue in the areas of the thighs and gluteal and abdominal region is still evident and reaches down to the muscle fascia. However, additional lipoatrophic lesions did not occur under ruxolitinib treatment and were no longer observed in the context of subcutaneous insulin injections. Interestingly, with the beginning of ruxolitinib treatment, the fatty tissue returned well in areas where insulin had not been injected before (Fig. 1c).

The primarily reported clinical course in patients with STAT3 GOF mutations is characterized by early lymphoproliferation and multiple autoimmune phenomena [3]. To date, 10 patients with activating germline STAT3 GOF mutations have been described with accompanying insulin-dependent diabetes, including six with neonatal-onset, as part of a poly-autoimmune syndrome (reviewed in supplementary Table 2). The majority of patients suffer from several manifestations simultaneously, including cytopenia, autoimmune enteritis, eczema, lymphocytic interstitial pneumonia, thyroiditis, arthritis, and autoimmune hepatitis [3].

STAT3 is one of seven transcription factors that contribute to signal transmission from cell membrane receptors to the nucleus together with Janus kinases (JAKs). After receptor stimulation by cytokine signaling, a JAK molecule recruits STAT to the receptor via receptor phosphorylation. This leads to STAT phosphorylation, dimerization, and translocation to the nucleus where effector genes are activated.

With JAK1/2-inhibitors, a targeted therapy is available that mitigates the course of immune-dysregulation in STAT3-GOF patients. Ruxolitinib is a small molecule that selectively blocks JAK1 and JAK2 and thus inhibits the activation of the JAK/STAT signaling pathway. Ruxolitinib has successfully been employed in these patients to treat severe immune dysregulatory phenomena. Its use must be carried out under close monitoring, as virus reactivations and increased susceptibility to viral infections may occur [2].

In our patient, the marked lipodystrophy may be interpreted as a potential inflammatory process of the subcutaneous fatty tissue, triggered by insulin injections, in the context of the STAT3-GOF mediated poly-autoimmune syndrome. This hypothesis is supported by the observation that those areas of our patient’s body treated with insulin beforehand continue to show partial lipodystrophy. Yet, despite ongoing insulin injections, no further development of lipoatrophic areas has been observed under ruxolitinib treatment. In addition to the disruption of an aberrantly high JAK/STAT-signaling induced inflammation by ruxolitinib, recent data also suggests that the regeneration of fatty tissue may be improved by leptin signaling blockade in the brain via JAK2/STAT3 [4].

Following our clinical observation and review of literature (supplementary Table 2), we highly recommend including *STAT3* in the genetic screening of patients with IDDM when the following criteria are observed:

1. Unusual (i.e., neonatal onset) manifestation
2. Multiple autoimmune phenomena, autoinflammation, or signs of lymphoproliferation
3. Unusual high demand of insulin
4. Pronounced lipoatrophy as the result of insulin injections

In conclusion, ruxolitinib medication may be beneficial to reduce autoinflammation and thereby also insulin demand in STAT3-GOF patients with (neonatal onset) diabetes. Ruxolitinib treatment of our patient was also associated with a marked decline of insulin antibodies (IAA). These patients require multiprofessional medical care, ideally in tight collaboration of immunologists and endocrinologists.

## Electronic supplementary material

ESM 1 (DOCX 33 kb)
