# Supplementary material for: Ruxolitinib Controls Lymphoproliferation and Diabetes in a STAT3-GOF Patient
Source: J Clin Immunol. 2020 Sep 17;40(8):1207–10. doi: 10.1007/s10875-020-00864-w (PMC7567728; doi:10.1007/s10875-020-00864-w)
Supplement: Supplementary file 1 — (DOCX 33 kb) [file 10875_2020_864_MOESM1_ESM.docx]

**Ruxolitinib controls lymphoproliferation and diabetes in a STAT3-GOF patient**

**Journal of Clinical Immunology**

Oliver Wegehaupt^1,2^, Tina Muckenhaupt^3^, Matthew B Johnson^4^, Karl O Schwab^1^, Carsten Speckmann^1,2*^

^1^Faculty of Medicine, Center for Pediatrics and Adolescent Medicine, Medical Center, University of Freiburg, Freiburg, Germany;

^2^Faculty of Medicine, Center for Chronic Immunodeficiency (CCI), Medical Center - University of Freiburg, Institute for Immunodeficiency, University of Freiburg, Freiburg, Germany;

^3^Diabetes Centre, Center for Pediatrics and Adolescent Medicine, Reutlingen, Germany;

^4^Institute of Biomedical and Clinical Science, University of Exeter Medical School, Exeter, United Kingdom.

*Correspondence:
Carsten Speckmann, MD; Center for Pediatrics and Adolescent Medicine, Medical Center, University of Freiburg; Mathildenstr. 1; 79106 Freiburg, Germany; Tel: +49-761-270-43000; carsten.speckmann@uniklinik-freiburg.de

**Supplementary Table 1** Features of patients with *STAT3* gain-of-function mutation p.Pro715Leu (c.2144C>T)

| No | Sex | Diabetes mellitus | Other immune dysregulation | Lymphoproliferation | Cytopenia | Short stature | Reference |
| --- | --- | --- | --- | --- | --- | --- | --- |
| 1 | M | + | Autoimmune thyroid disease | Generalized lymphoproliferation | Thrombopenia, neutropenia | + | Sediva et al. Horm Res Paediatr 2017 |
| 2 | F | - | - | Hepatosplenomegaly, generalized lymphoproliferation | Thrombopenia, anaemia | + | Sediva et al. Horm Res Paediatr 2017 |
| 3 | M | - | Hashimoto thyroiditis, eczematous dermatitis | Splenomegaly, diffuse lymphadenopathy | Thrombopenia | + | Forbes et al. JACI 2018 |
| 4 | NA | - | Bilateral keratoconjonctivitis | Hepatosplenomegaly | Thrombopenia | NA | Besnard et al. Clin. Immunol. 2018 |
| 5 | M | + | panniculitis | Inguinal and abdominal lymphadenopathy | - | - | Wegehaupt et al. 2020 |

*Abbreviations:* NA: data not available; f: female; m: male

**Supplementary Table 2** Patients described with *STAT3* gain-of-function mutations and diabetes

We searched PubMed for articles published in English and examined original articles, case reports, case series, and review articles. The following terms were used in various combinations: “STAT3”; “diabetes”; “neonatal diabetes”; “neonatal onset diabetes”; “neonatal diabetes monogenic”. Cross-references were checked and duplicates were sorted out.

| **No** | **Sex** | **Current age (years)** | **Birth weight (SDS)** | **Growth (Height SDS)** | **Diagnosis of Diabetes** | **Autoantibodies** | **Pancreatic exocrine insufficiency** | **Current insulin dose (IE/kg/d)** | **Current HbA1c (mmol/mol)** | **Type and effect of mutation** | **Reference** |
| --- | --- | --- | --- | --- | --- | --- | --- | --- | --- | --- | --- |
| **Neonatal / Infant onset** | | | | | | | | | | | |
| **1** | F | 6 | - 1.59 | - 2.33 | 2 wks | GAD65 | Fecal elastase  166 mg/g faeces (>200) | 1.8 | 61 | p.Thr716Met  c.2147C>T | Flanagan et al. Nature Genetics 2014 |
| **2** | F | 15 | - 5.81 | - 6.63 | 0 | GAD65, IAA, ICA | On enzyme  replacement therapy-  Low Fecal elastase | 1.5 | 70 | p.Lys392Arg  c.1175A>G | Flanagan et al. Nature Genetics 2014 |
| **3** | M | 6 | - 2.7 | - 1.47 | 3 wks | Not elevated | NO | 0.76 | 68 | p.Asn646Lys  c.1938C>G | Flanagan et al. Nature Genetics 2014 |
| **4** | M | 3 | - 1.59 | - 2.05 | 43 wks | GAD65, IA-2 | NO | 0.8 | 69 | p.Asn646Lys  c.1938C>G | Flanagan et al. Nature Genetics 2014 |
| **5** | M | 5,5 | NA | NA | 11 mo | NA | NA | NA | NA | p.Pro715Leu c.2144C>T | Sediva et al. Horm Res Paediatr 2017; same patient in Strakova et al. 2019 |
| **6** | F | NA | NA | - 3 (final height) | 3 mo | ICA | NA | NA | NA | p.Pro330Ser c.988C>T; | Velayos et al. Diabetes 2017 |
| **7** | M | 2 | + 2.9 | + 0.3 | 3 mo | IAA, GAD | NO | 0.6 | 51 | p.Pro715Leu c.2144C>T | Wegehaupt et al. 2020 |
| Five additional patients have recently been identified in Exeter, U.K. (A. Hattersley and M. Johnson, personal communication). | | | | | | | | | | | Fabbri et al. Diabetes 2019 |
| **Childhood onset** | | | | | | | | | | | |
| **8** | M | 25 | NA | NA | 3 yrs | NA | NA | NA | NA | p.R152W | Milner et al. Blood 2015 |
| **9** | F | 9 | NA | NA | 5 yrs | NA | NA | NA | NA | p.E415K | Milner et al. Blood 2015 |
| **10** | M | 18 | NA | NA | 3 yrs | NA | NA | NA | NA | p.F313L (c.937T>C) | Maffucci et al. Frontiers 2016 |
| **11** | F | NA | NA | NA | 2 yrs | NA | NA | NA | NA | M394T | Nabhani et al. Clin Immunology 2017 |

*Abbreviations:* NA: data not available; f: female; m: male; diagn: diagnosis; wks: weeks; mo: months; yrs: years
